# Revision of the Failed Cleft Lift for Pilonidal Disease

**DOI:** 10.7759/cureus.34511

**Published:** 2023-02-01

**Authors:** Steven C Immerman

**Affiliations:** 1 Surgery, Evergreen Surgical, Eau Claire, USA

**Keywords:** perianal pilonidal wounds, cleft lift technique, failed pilonidal surgery, bascom cleft lift, cleft lift surgery, hidradenitis suppurative, chronic pilonidal disease, pilonidal cyst surgery, pilonidal sinus surgery, pilonidal

## Abstract

The cleft lift has been demonstrated to be one of the most successful operations for the treatment of pilonidal disease, however, there are times this procedure fails and further surgery is necessary. This article describes a reproducible and successful technique for the revision of a failed cleft lift. This procedure was performed on 76 consecutive patients who had previous cleft lift procedures. Failures were manifested by either a wound, sinus, abscess, dehiscence or fragile scar. The revision flattened the lower gluteal cleft with a rotation and advancement flap that placed the skin incision off-midline. Follow-up over the 10 years of this series was between six and 124 months with an average of 36 months. The revision was initially successful in 96.1% of patients; if the procedure was unsuccessful a repeat revision was subsequently curative. This procedure is proposed as an essential part of the treatment algorithm for patients with recurrent pilonidal disease after a cleft lift operation.

## Introduction

The Bascom Cleft Lift has been proposed as a salvage procedure after the failure of most other pilonidal operations as well as an appropriate surgical strategy for the treatment of primary pilonidal disease [1-3]. Although in experienced hands the Bascom Cleft Lift and Karydakis Flaps have been shown to have the lowest long-term failure rates of any of the described procedures to treat pilonidal disease, failures do occasionally occur [4]. The failure can be manifested by either the initial failure of the incision to completely heal or by recurrent pilonidal disease that appears after primary healing. It is important that when failures occur with any operation for pilonidal disease that there are subsequent steps in the treatment algorithm such that patients are cared for until complete and durable healing occurs. This article discusses one of the important advantages of the cleft lift procedure, which is that failures can be surgically treated by revising the shape of the residual cleft with a high chance of success.

## Materials and methods

This is a series of cleft lift revisions over a 10-year period. Patients were accrued consecutively between 2012 and 2022 and no patients with recurrent pilonidal disease following a cleft lift were excluded during this time frame. Current follow-up was obtained by either email survey, phone conversation, or review of current electronic medical records. If a patient required a second revision, the length of follow-up was calculated starting after the second revision.

Technique

After the administration of general anesthesia, the patient is placed prone and flat on the operating table with a slight Trendelenburg tilt. Intravenous antibiotics are administered consisting of ciprofloxacin and metronidazole, or ampicillin/sulbactam unless patient allergies required different choices. No preoperative bowel preparation was performed.

**Figure 1:**
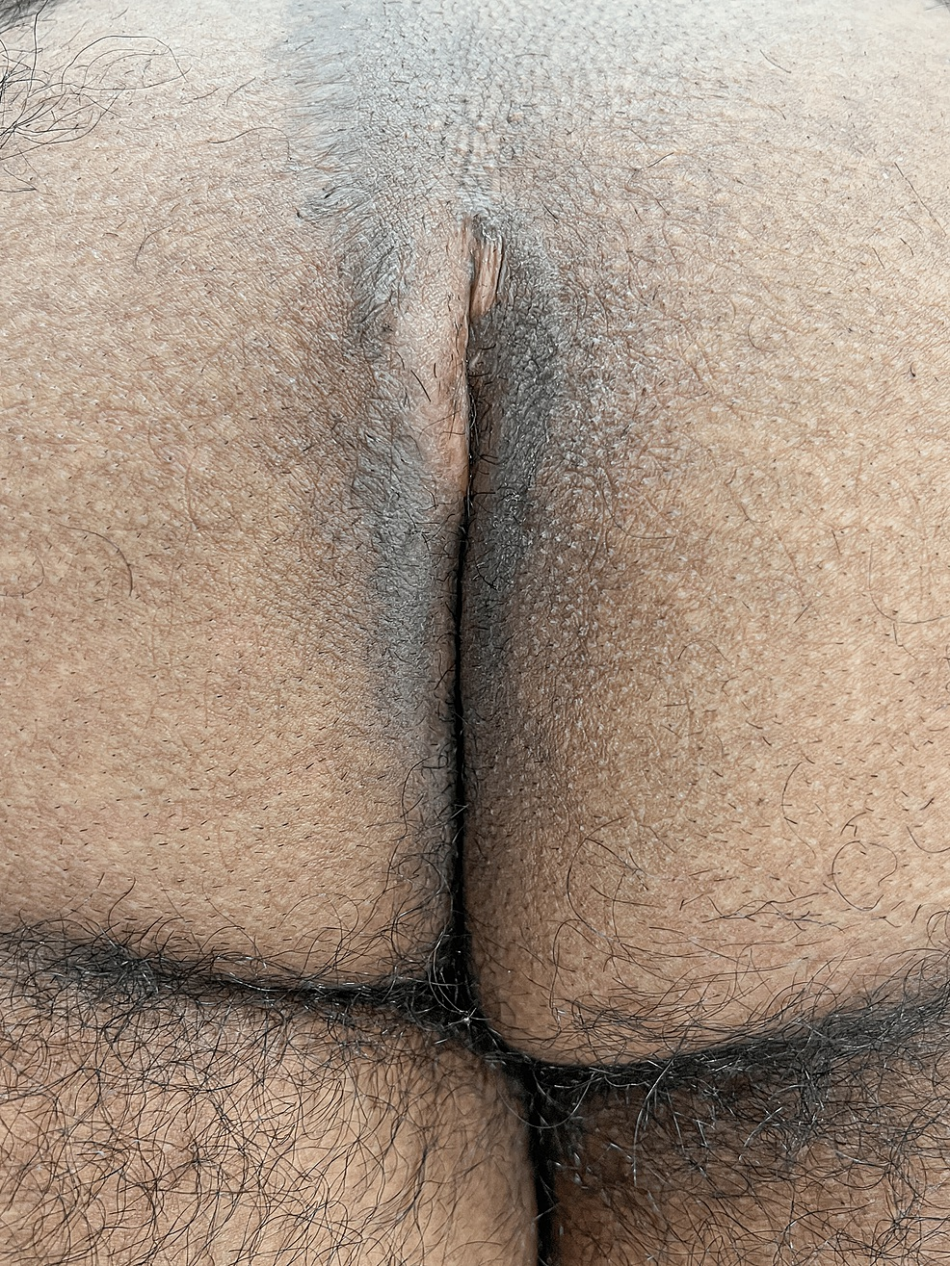
This patient has a deep perianal cleft with an open wound that developed six months after a cleft lift. Note the cleft lift scar to the left of the midline.

**Figure 2:**
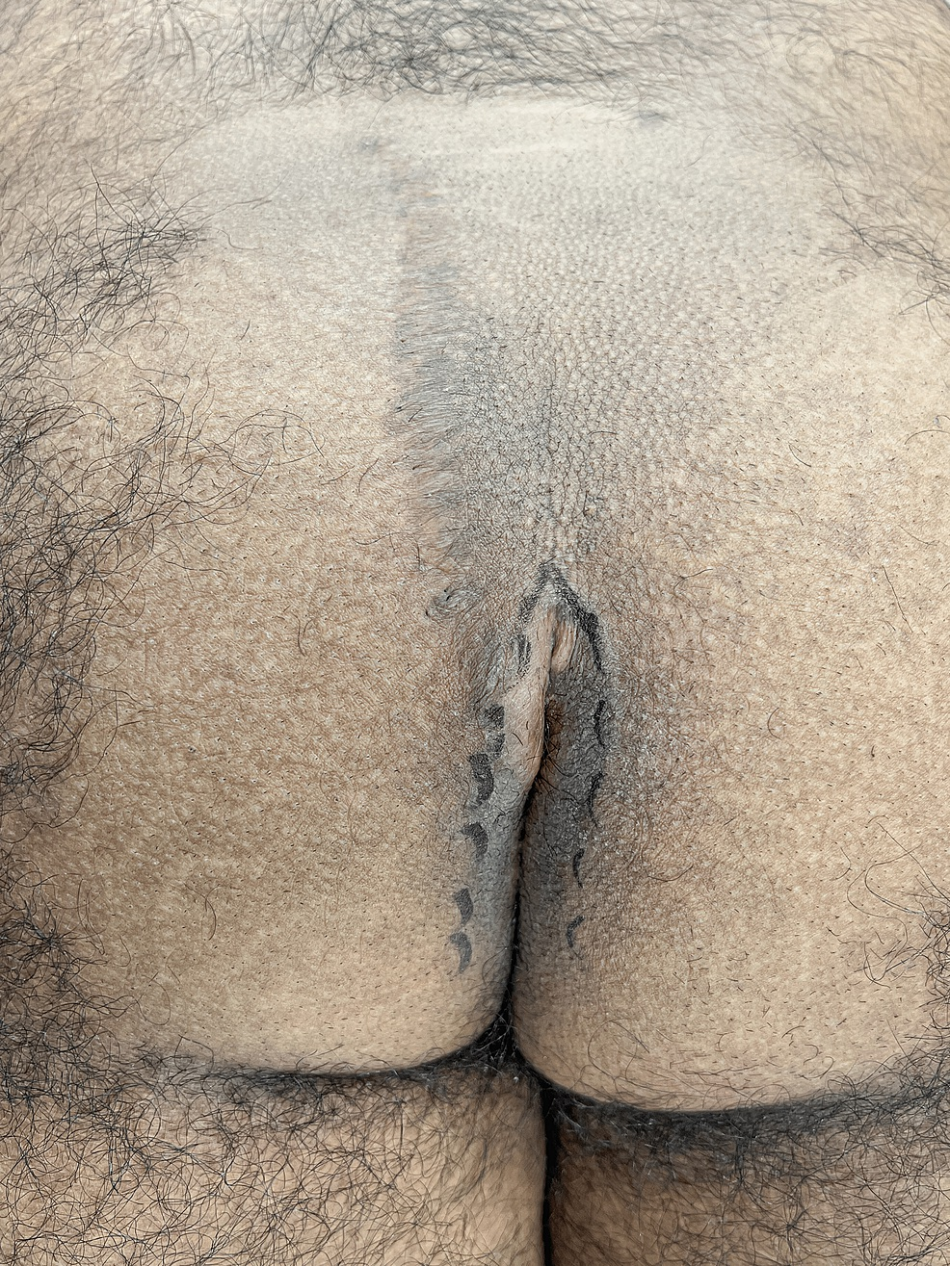
The line of contact between the gluteal cheeks has been marked with a dotted line.

The buttocks are compressed medially, and the line of contact between the two sides of the gluteal cleft is marked with a dotted line for future reference (Figures 1-2).

**Figure 3:**
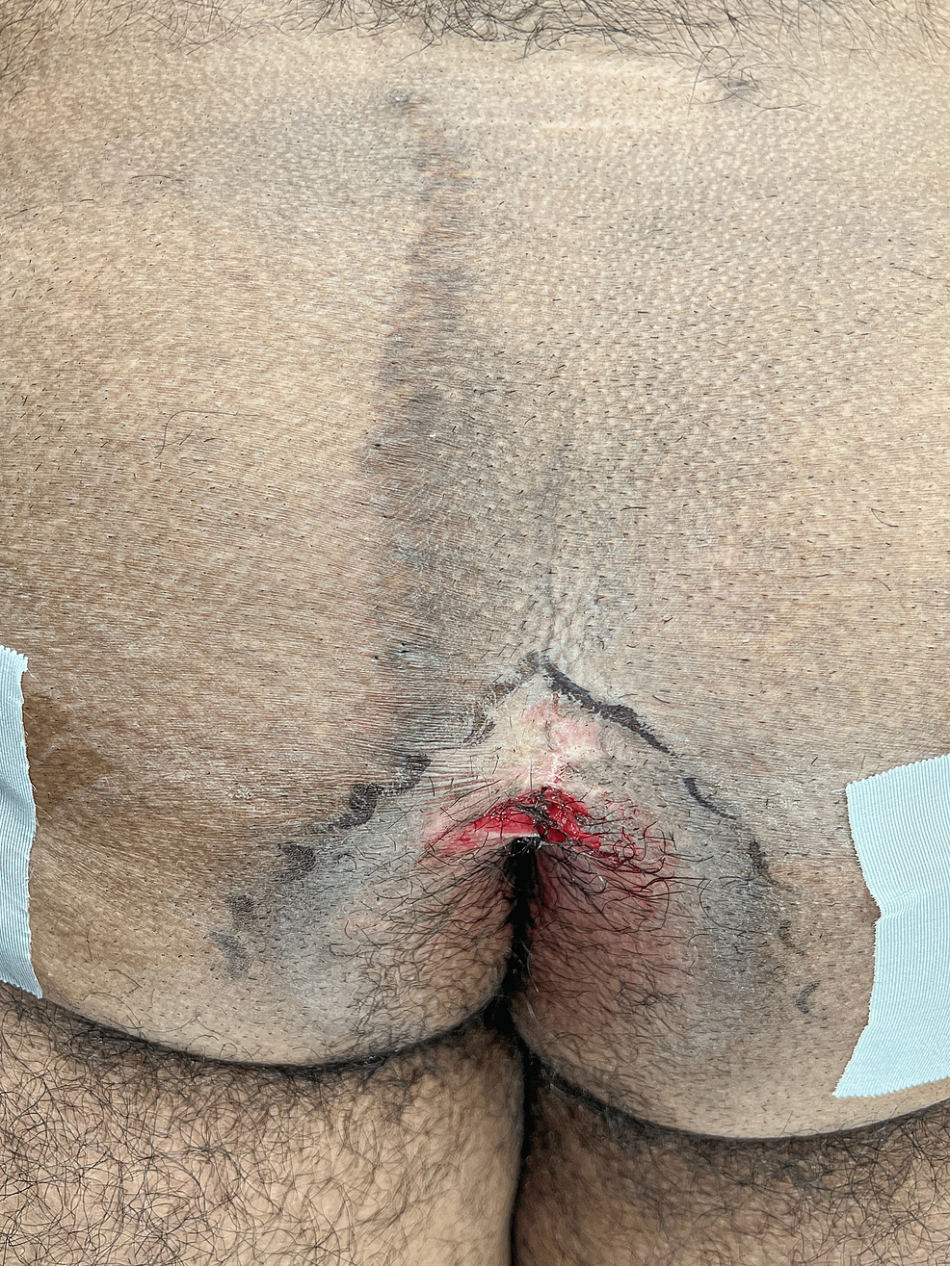
The buttocks are taped apart and the wound now becomes evident.

This line of contact may exist only in the caudal end of the previous incision because of the flattening accomplished by the previous cleft lift. Once the line of contact has been marked the buttocks are taped apart to expose the anus (Figure 3).

The operative site is now clipped of hairs, and the skin is marked with the proposed area of excision, as described in the next step. After the marking is performed, the skin is both scrubbed and painted with a povidone-iodine antiseptic.

**Figure 4:**
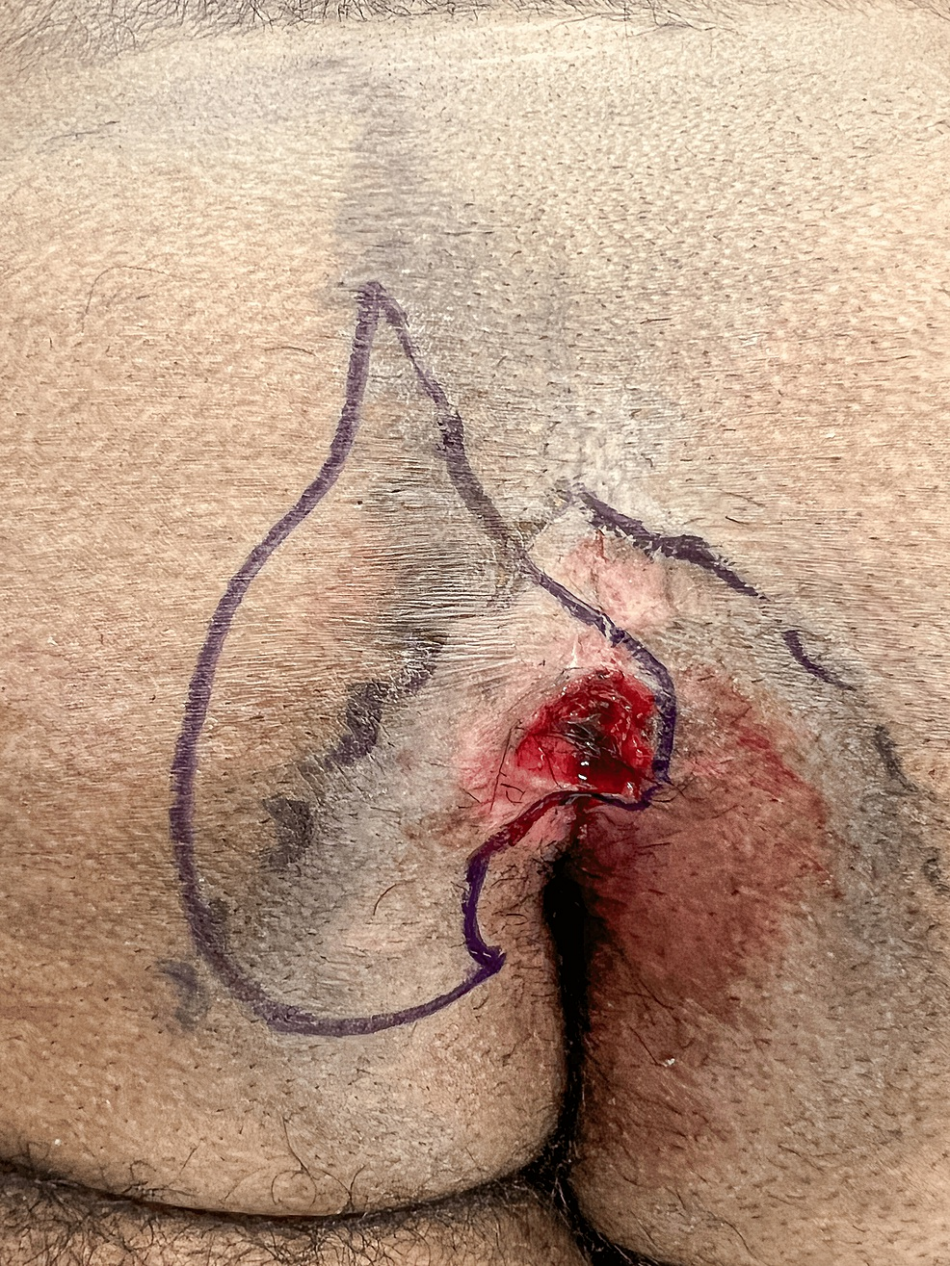
Preliminary markings are made in preparation for the medial incision. The lateral markings will be modified as the procedure progresses.

The medial limb of the incision is marked by following the lower part of the scar from the previous cleft lift. As the line extends inferiorly it curves around the lowermost wound (or primary sinus tract opening) and then curves onto the opposite side as shown in Figure 4. How far cephalad this incision begins is dependent upon how much residual cleft exists superiorly, whether there are any secondary sinus openings in that area, how much mobility is needed, and whether there seems to be some value to removing the upper portion of the scar.

The lateral limb of the incision is then marked, with the understanding that when making the actual incision this may be modified. This lateral marking is an arc extending from the previous scar, and approaching the anus from the 3 or 9 o’clock position, depending on the operative side. This always extends laterally to the dotted line of contact. If an attempt is made to stay medial to the line of contact the result will be inadequate to accomplish flattening the cleft and positioning the incision off-midline. The lines marking the proposed incision are infiltrated with 0.5% bupivacaine with epinephrine.

**Figure 5:**
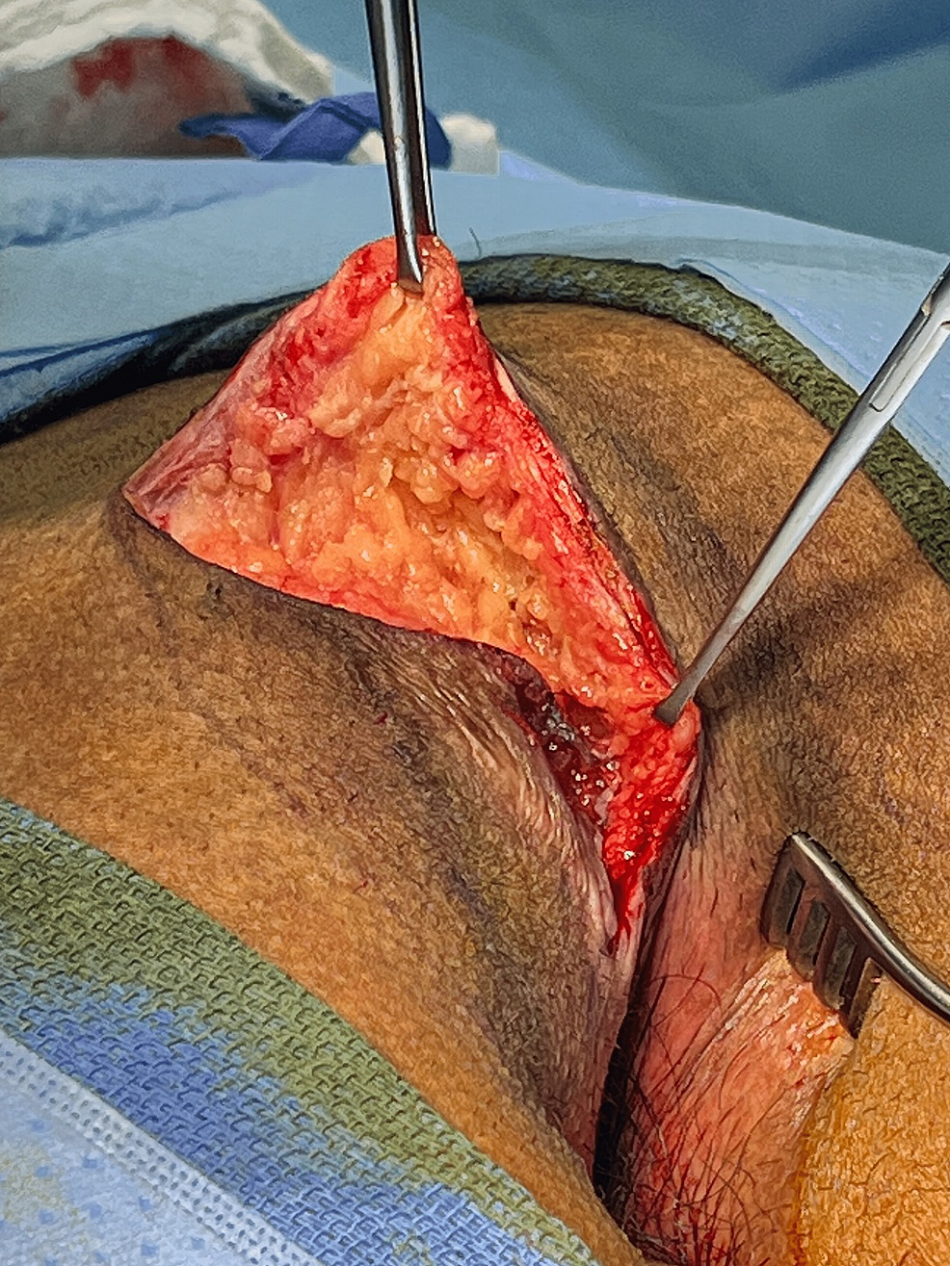
The flap is displayed by retraction with Allis clamps.

The medial portion of this incision is made with a scalpel. The incision follows the marked line from the cephalad part of the marking down and around the midline wound and about 2 cm past the midline. From this cut edge, a skin flap is raised (on the same side as the flap was raised in the previous cleft lift) with a thickness of 1 cm or slightly less (Figure 5). The flap is raised approximately to the line of contact. Additional mobilization may be performed later in the procedure if necessary. Raising the flap more than necessary or making it thicker is not beneficial. It is important to carefully develop the flap inferiorly toward the anus, loosening any scar tissue or tethering, to gain mobility for rotation of the flap. Care is taken to avoid making this section of the flap too thin, and if necessary some of the external anal sphincter fibers can be left on the flap.

**Figure 6:**
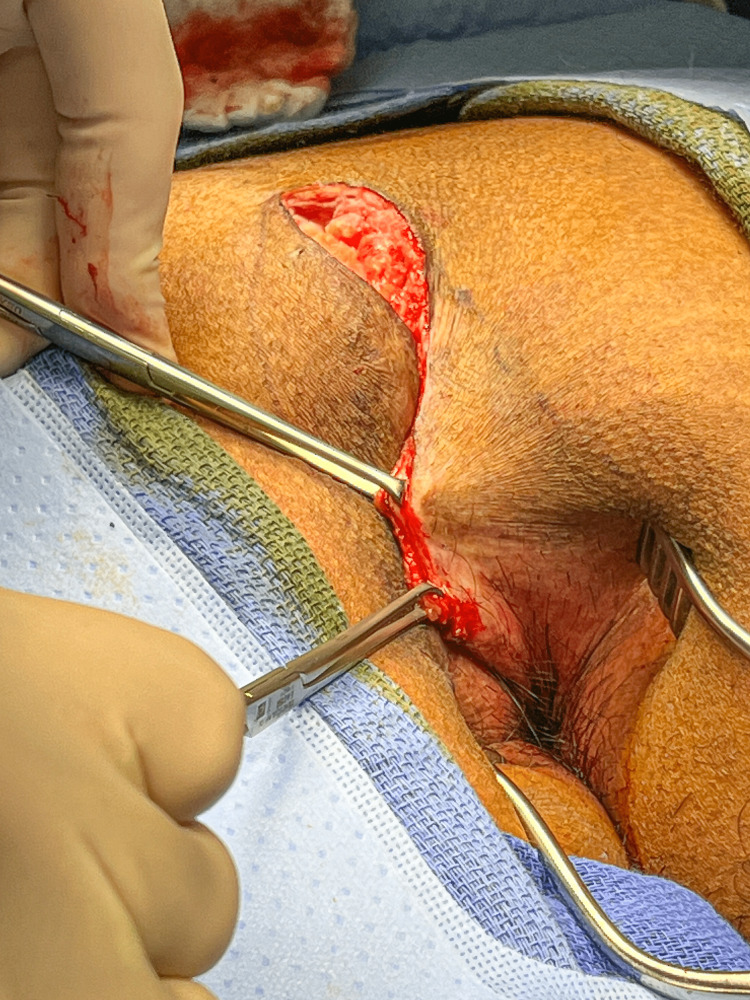
With the tapes removed, the lower flap is rotated to the left in this patient, demonstrating how a fold develops in the 10-11 o'clock position, pointing toward the anus.

Once the flap has been raised, the tapes on the buttocks are removed, and the flap is pulled across the midline to check mobility using Allis clamps on the deep dermis (Figure 6).

**Figure 7:**
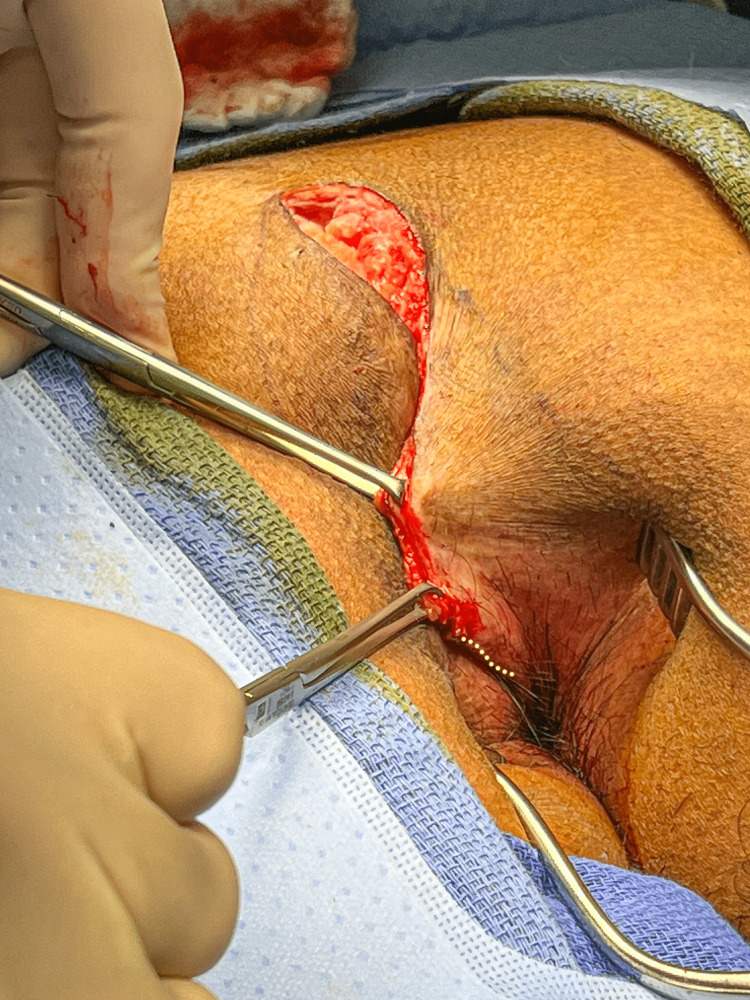
The fold demonstrated in Figure 6 is marked in preparation for an incision, as indicated by the yellow dotted line.

Inferiorly, the skin can be seen to fold, with the fold pointing radially toward the center of the anus. The line of this fold is marked as demonstrated by the dotted line in Figure 7.

**Figure 8:**
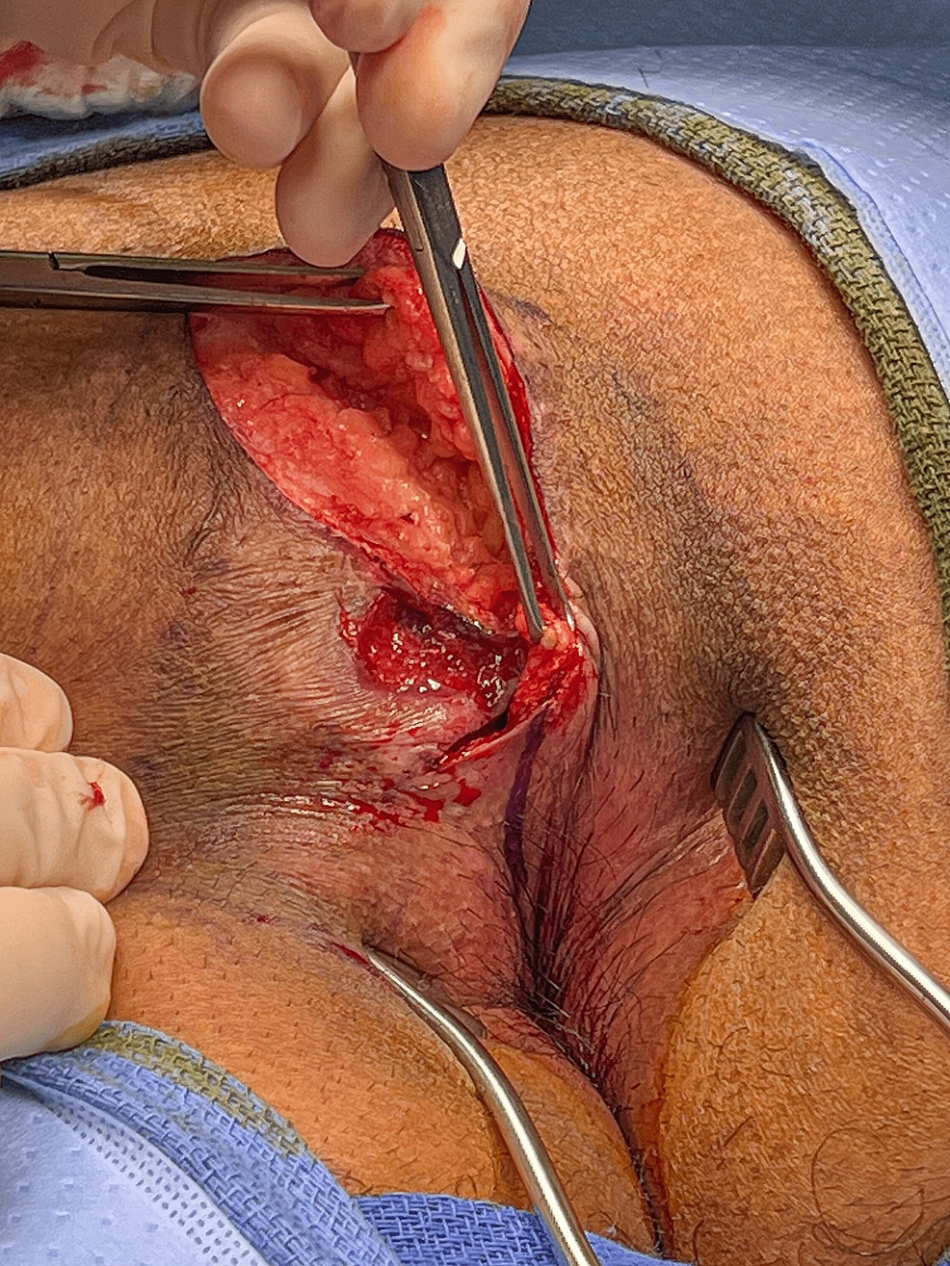
This image demonstrates the marking is defined by the fold demonstrated in Figure 7. An incision is made on this line. Note that it comes to the outer edge of the anoderm, but does not enter the anal canal.

An incision is made along this line which was marked by a blue surgical marker in Figure 8.

Once the medial incision is completed, the next step is to check if the *lateral* markings are optimal for the current situation. Video 1 shows a different, but similar, patient and demonstrates how the perianal part of the *lateral* incision is marked. In this patient, the incision extends significantly higher because of a sinus tract extending to the upper end of the previous cleft lift incision.

**Video 1:** Once the medial incision is made and the tapes removed, the lower part of the flap is rotated laterally and inferiorly to define the lower extent of the lateral flap.

The lateral part of the incision should be well off the midline without creating undue tension on the closure. Video 2 demonstrates bringing the flap across the midline and evaluating tension. It is acceptable to slightly push the buttocks together as this is evaluated; finding this balance is acquired with experience.

**Video 2:** The entire flap is brought across the midline to check position and tension.

**Figure 9:**
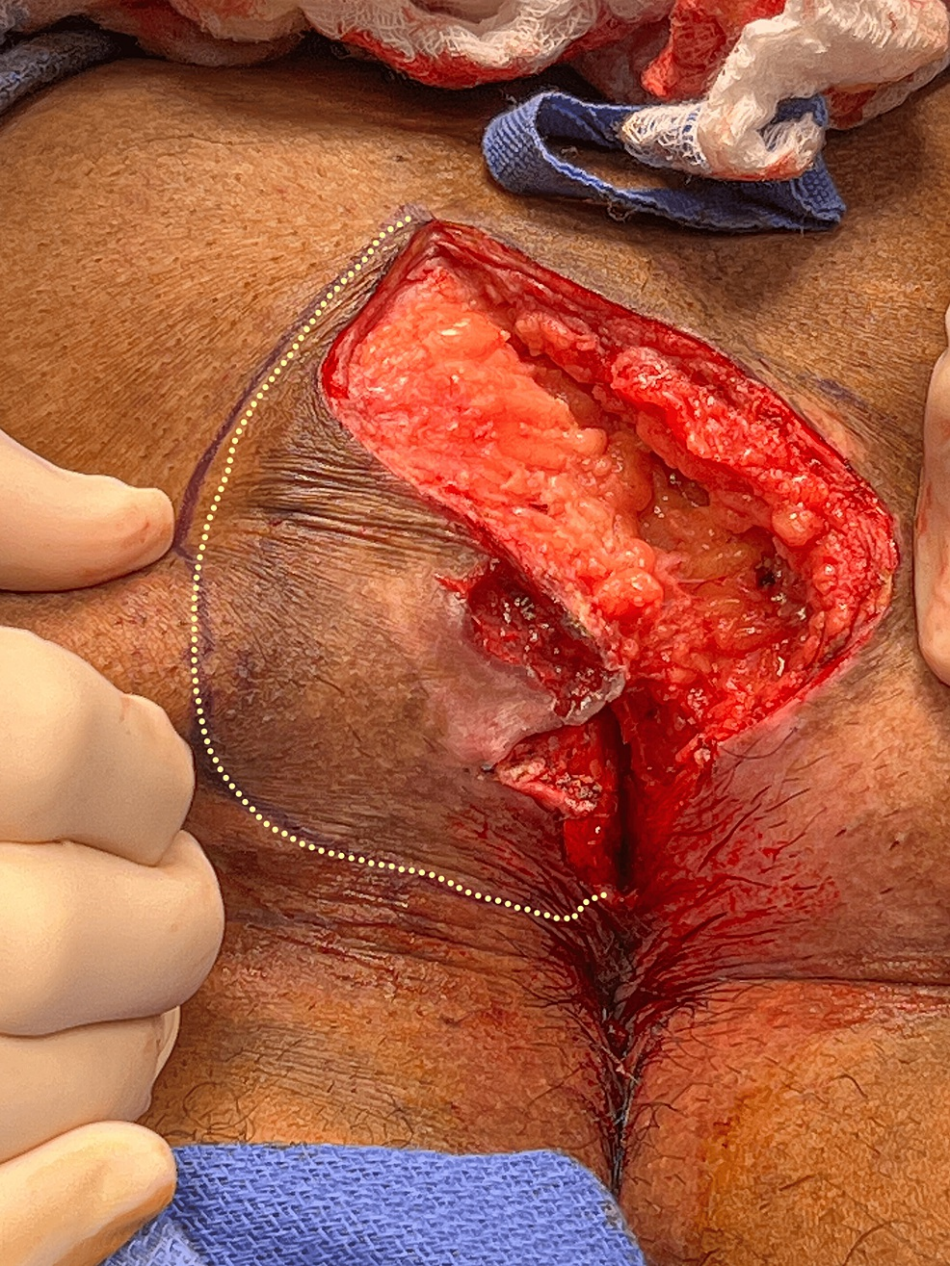
The dotted line demonstrates the lateral incision in this patient. The excision will remove the skin on the left side of the cleft and the midline disease.

The lateral line is adjusted if the flap reaches farther, or not as far, as originally marked. This new marking will define the second side of the excision. In general, the removal of 4 cm or more of skin on the side opposite the flap will result in an incision that is satisfactorily away from the midline, but this needs to be evaluated as the procedure proceeds and modified if needed. Figure 9 shows the marking for the lateral incision in this patient.

**Figure 10:**
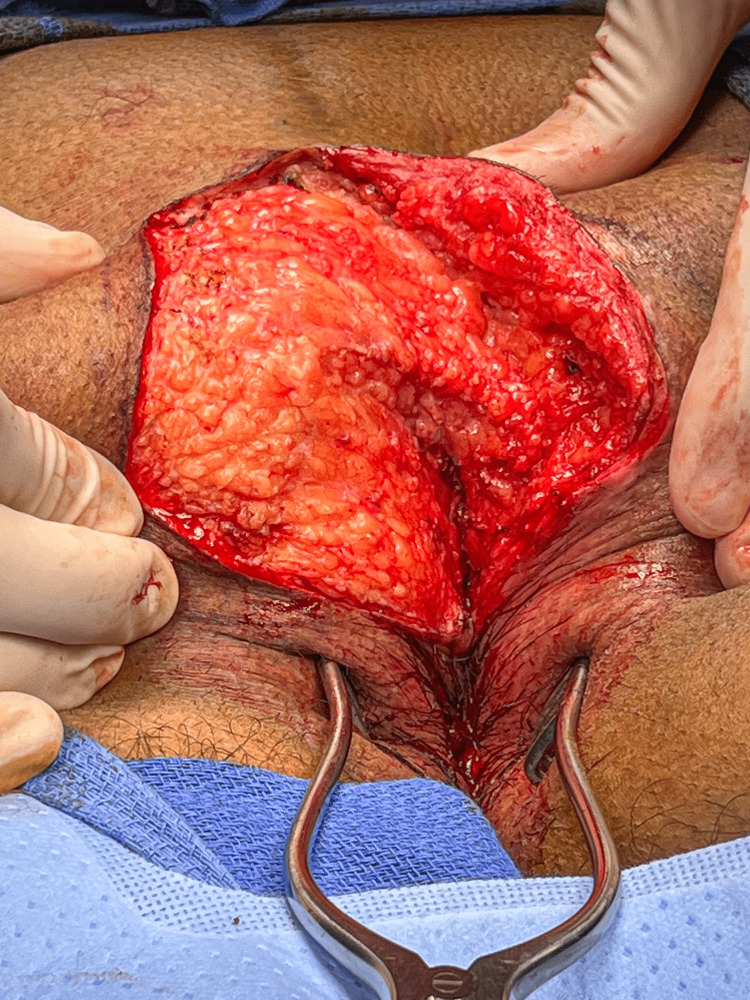
The skin on the left side of the cleft has been removed along with the midline disease. Here we see the clean wound bed and raised flap.

The lateral incision is then made and the skin on the non-flap side of the cleft, along with the midline disease and sinus tracts, is removed. Care is taken to remove as little subcutaneous tissue as possible while removing any areas of granulation tissue, hair, and scar. The appearance of the wound after skin removal is shown in Figure 10. Note that the incision does not enter the anal canal, and is kept about 1-2 cm from the anal opening.

The wound is irrigated with sodium oxychlorosene antiseptic solution (Chlorpactin™), infiltrated with liposomal Marcaine (Exparel™), and might be drained with a 15 French channel Drain (Blake™) depending on how far the excision ends superiorly. Frequently, a drain is not necessary if the area of dissection is limited to the perianal area of the cleft. If a drain is placed, it is brought out on the flap side, but lateral and superior to the flap.

**Figure 11:**
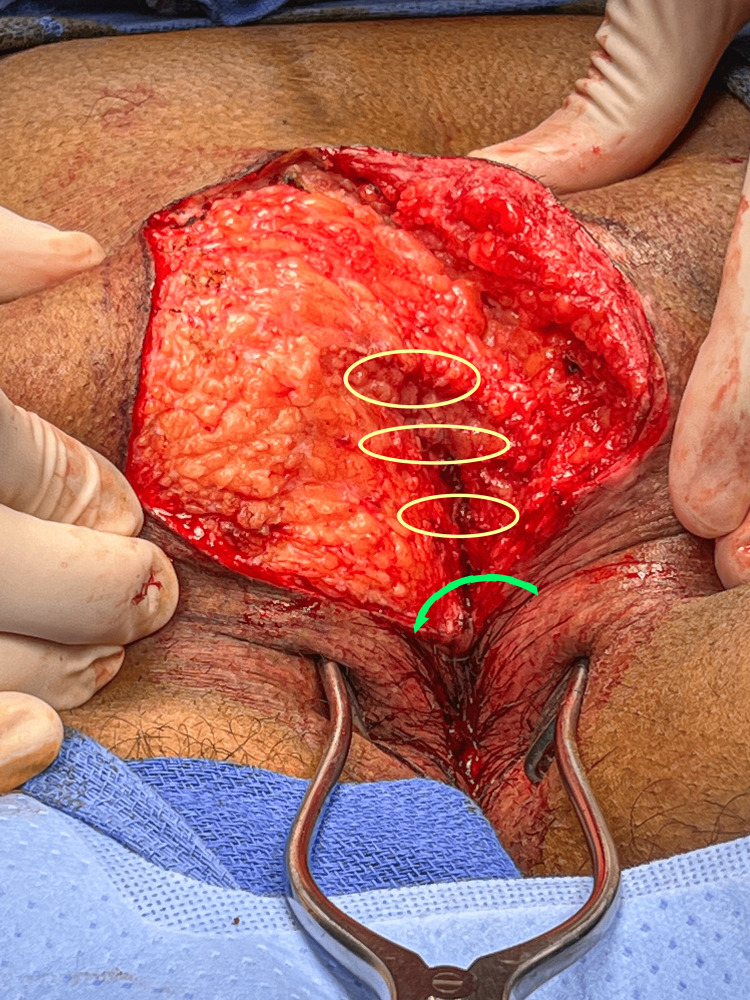
The midline subcutaneous tissue is approximated with sutures in the locations of the elliptical illustrations. The flap will be rotated as indicated by the green arrow in the next step.

The midline subcutaneous fat is sutured together with sutures of 2-0, monofilament, absorbable, figure-of-eight sutures. Closure of this layer eliminates dead space, prevents the development of a deep cleft, and assists in lateralization of the incision. The yellow elliptical markings in Figure 11 demonstrate the placement of these sutures, and the green arrow shows the direction that this will be closed.

**Figure 12:**
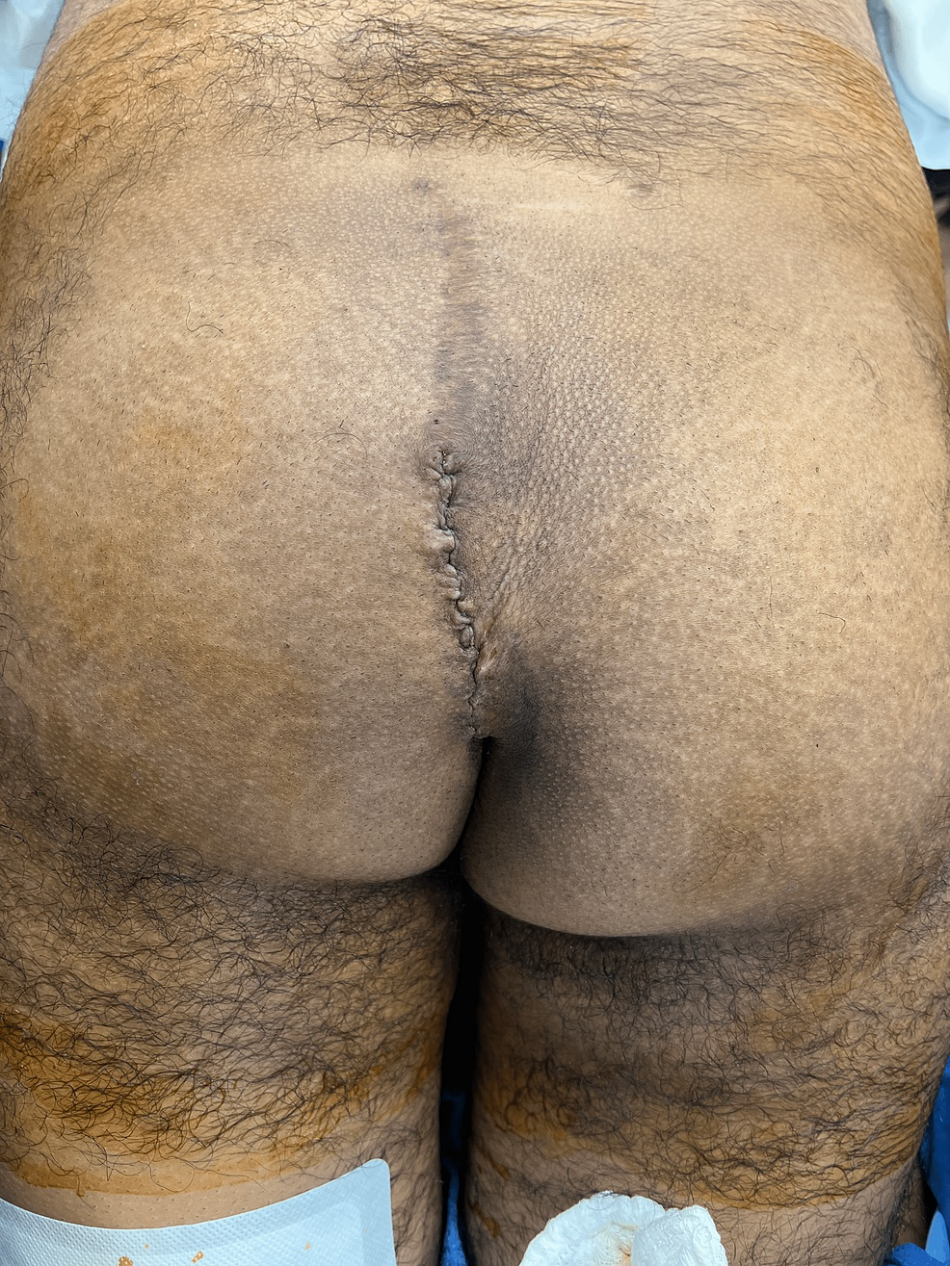
The completed cleft lift revision after skin closure.

The flap is brought across the midline and sutured in place with interrupted 2-0, absorbable, monofilament suture in the deeper layers, and interrupted, 3-0, absorbable, monofilament suture in the deep dermis with all knots buried in both layers. The closure must start at the inferior end of the incision and proceed superiorly, with an attempt to nestle the medial skin edge into the curve of the lateral skin edge. As closure proceeds, periodically pushing the buttocks together to visualize the location of the newly created gluteal fold is beneficial in evaluating the lateralization of the incision. If during this maneuver the incision is found to be in the midline and in a fold, additional tailoring of the shape is required by removing more skin on the non-flap side and attempting closure again. With wounds directly on the edge of the anus associated with significant scarring, it may not be possible to lateralize the part of the incision overlapping the anoderm. Once it is felt that the shape and position of the incision are satisfactory, the skin is closed with a running, subcuticular, monofilament suture. At the time of this writing, the author prefers polyglyconate (Maxon™) in the three deeper layers because of its prolonged strength profile, and a unidirectional poliglecaprone 25 (Stratafix Spiral Monocryl Plus™) in the skin, but 2-0 poliglecaprone 25 (Monocryl™) was used in all layers on the majority of these patients with excellent results. Figure 12 shows the completed closure.

**Figure 13:**
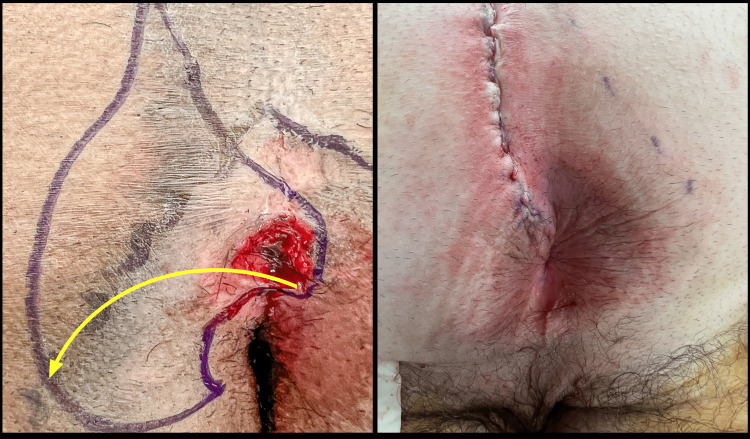
The initial marking compared to the final incision in the perianal area. Note how the final incision approaches the anus off-midline.

The right-sided image in Figure 13 shows the angle with which the incision approaches the anus and compares it to the original markings shown on the left. This procedure rotates the perianal tissue in such a way as to move the incision from one side to the other. Because of the natural redundancy of perianal tissue, this is almost always possible. In this case, even though the initial incision extended down the cleft toward the anus in the 2 o'clock position, the counterclockwise rotation brought the final location of the incision to the 11 o'clock position. In the photo on the right, if one observes the radially oriented folds in the perianal tissue, one can see that between the 2 and 11 o'clock position, there are relatively few folds because this rotation flattened the tissues.

**Figure 14:**
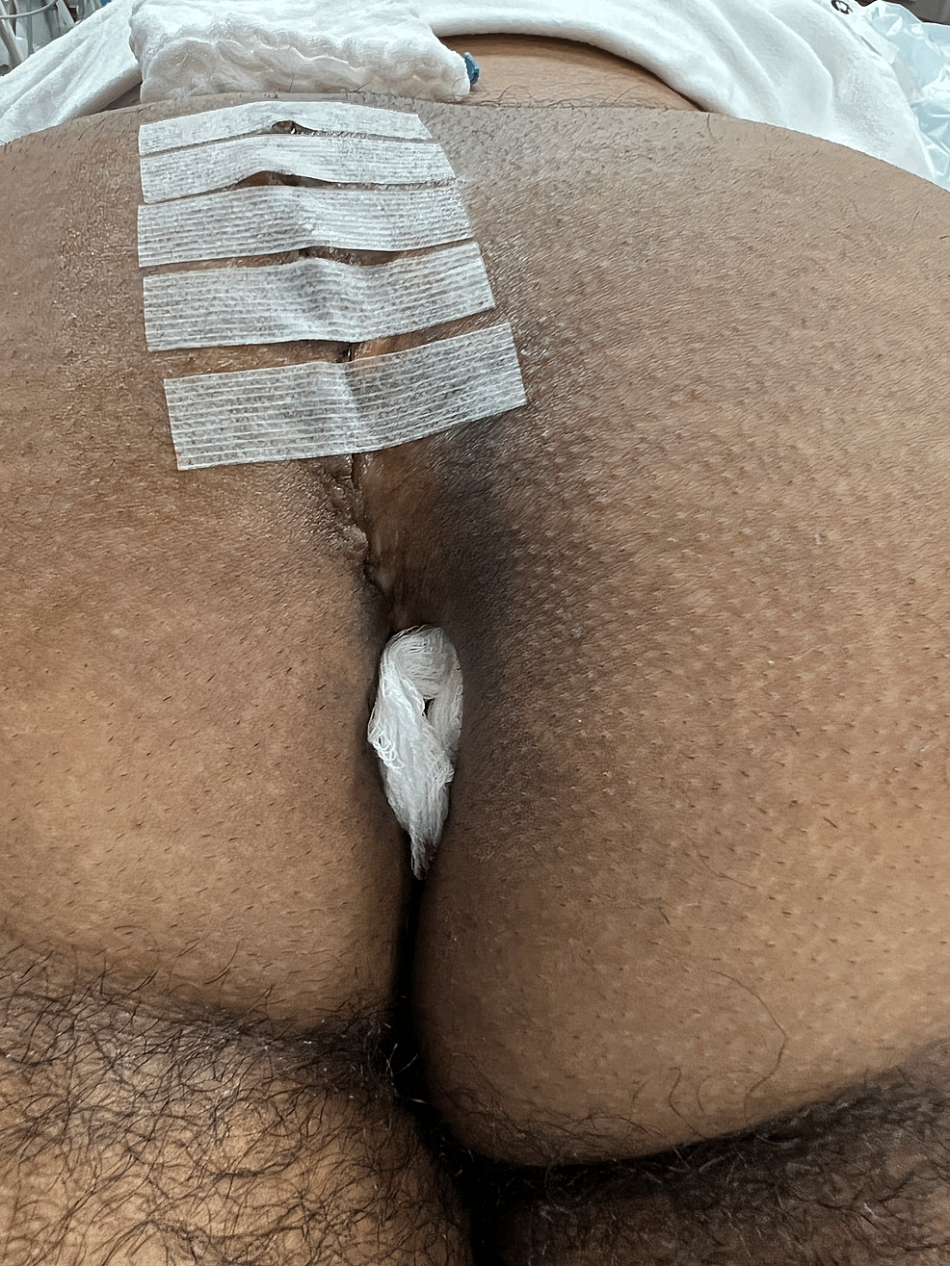
Steri-Strips™ are placed on the upper portion of the incision, and woven gauze is placed adjacent to the perianal limb of the incision to promote aeration.

Benzoin and Steri-Strips™ are applied to the portion of the wound superior to the anus, and a folded piece of woven gauze is tucked next to the lower limb of the incision (Figure 14).

**Figure 15:**
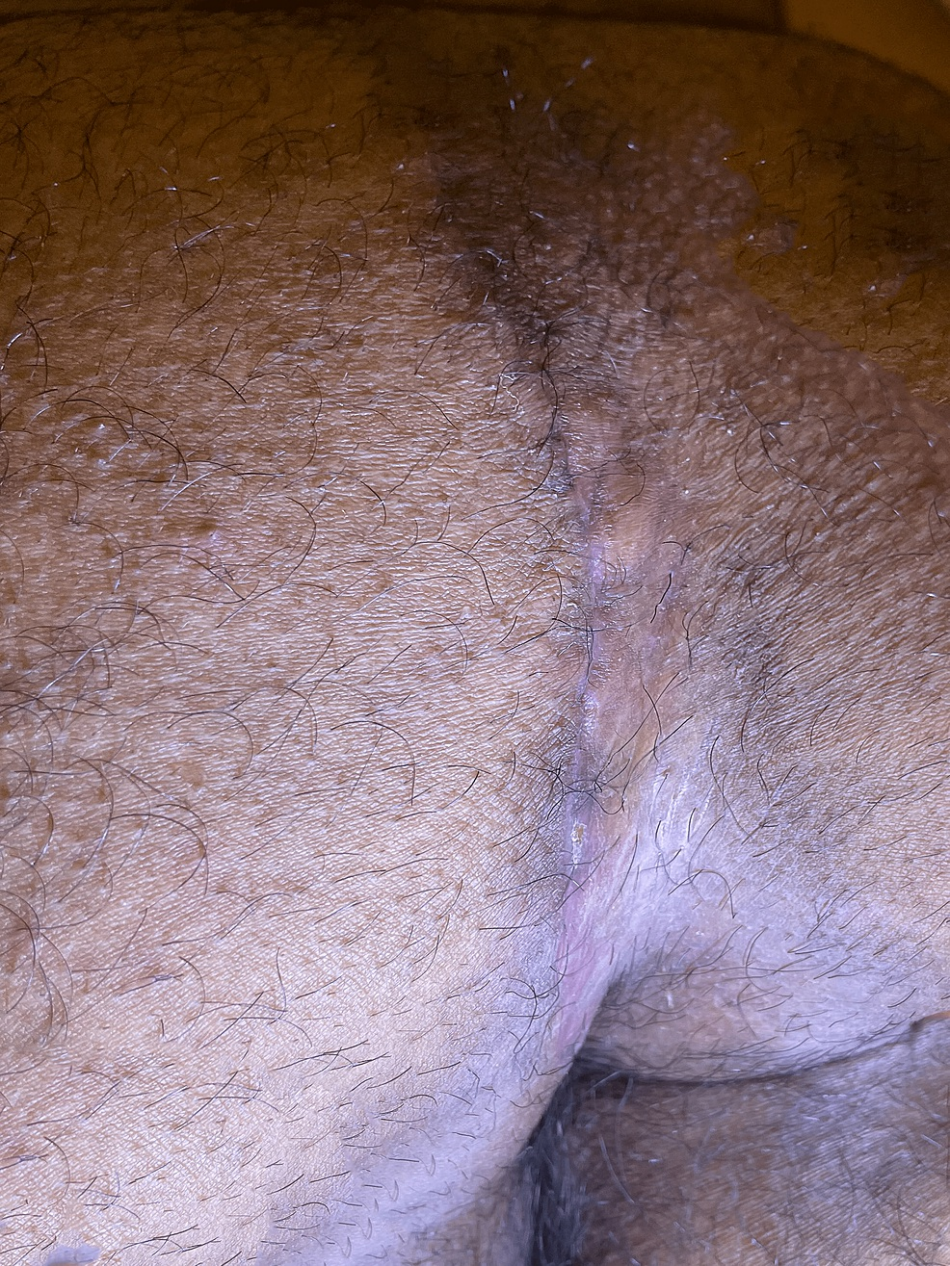
The appearance at three months postoperative.

**Figure 16:**
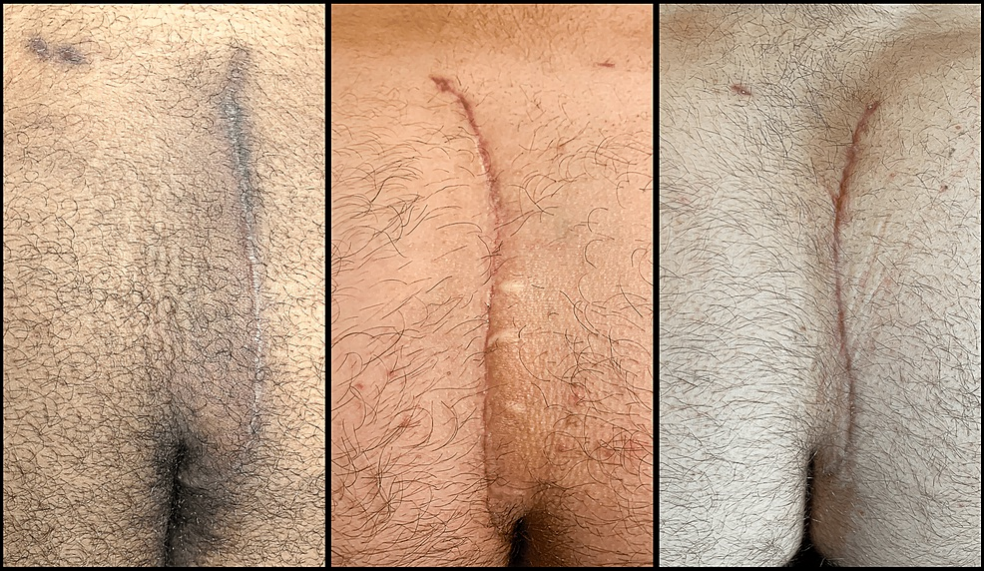
Examples of three other patients in this series, each approximately six weeks postoperative.

If a drain is placed, it is removed once the output was equal to or less than 20 cc/day, as long as at least five postop days had elapsed. Figure 15 shows the appearance 3 months after the revision, and Figure 16 shows the postoperative appearance of three other patients in this series approximately 6 weeks after surgery.

Postoperative Care

Patients are asked to avoid trauma to the incision for six weeks by avoiding contact sports, biking, or jogging. Sitting is encouraged immediately after the operation. No postoperative bowel regimen is used, but stool softeners are recommended as needed. Patients are allowed to shower the day after surgery but asked to avoid prolonged soaking in a tub or pool for four weeks. Dressings over the incision and drain site are changed daily to keep the incision clean and dry for about five days. All patients are asked to tuck a folded 4"x4" piece of woven gauze next to the perianal limb of the incision to allow air circulation. This is changed at least twice daily and continued for at least four weeks. Steri-Strips™ are removed two weeks postoperatively. Postoperative ciprofloxacin and metronidazole or ampicillin/clavulanate are given for one week. Patients are encouraged to use acetaminophen or ibuprofen for pain, but an additional narcotic is often also prescribed, such as tramadol or hydrocodone. Patients were not instructed to perform any type of hair removal after the operation.

## Results

There were 76 patients in this series; 22 of these were failures of the author’s cleft lift operations and 54 were failures of other surgeons’ cleft lift and Karydakis Flap procedures. The average age of the patients was 24 years, with a range of 14-70 years. These patients each had at least one previous operation for pilonidal disease (i.e., the cleft lift) before this revision. However, many had other prior procedures as well with an average of 2.4 previous procedures per patient in total, with a maximum of eight total procedures before the revision (Table 1).

**Table 1:** Cohort characteristics.

	Number	Percent
Male	53	70
Female	23	30
Average age	24	
Average prior operations	2.4	
Previous cleft lift	72	
Previous Karydakis Flap	4	
Previous additional complex flaps	3	
Edge of anus wounds	26	34

All procedures to eradicate pilonidal disease are tabulated except incision and drainage for acute abscess. Among this group, there are several patients who had complex flap procedures in addition to excisional procedures and a cleft lift. This included one patient each with bilateral gluteal muscle flaps, unilateral VY-Plasty, and bilateral VY-Plasty. Of the total group, four had failed Karydakis flaps.

Of the 76 patients, 26 (34%) had wounds that were not only in the perianal region (within 5 cm of the center of the anus) but actually reached the edge of the anoderm, and are in the category that we refer to as “edge of anus” lesions. The anoderm is defined as the area between the dentate line and the anal verge composed of nonkeratinized squamous epithelium and without hair follicles.

Current follow-up was obtained on 71 (92%) of the patients by either email survey, phone conversation, or review of current electronic medical records. This series extended back 10 years, and even if current follow-up was not possible, most patients were followed for several years after their revision with an average length of follow-up of 36 months, ranging from 124 months for the first patient of the series, to six months for the most recent patient.

The indication for revision was a persistent or recurrent wound without sinus tract formation (N=41), a sinus tract with a primary and secondary opening (N=28), postoperative wound dehiscence (N=2), recurrent abscess formation (N=3), or a fragile scar that intermittently separated and healed (N=2) (Table 2).

**Table 2:** Indications for cleft lift revision.

	Number	Percent
Wound	41	54
Sinus tract	28	37
Recurrent abscesses	3	4
Post op dehiscence	2	2.5
Fragile scar	2	2.5

The consistent physical finding was that all patients still had a residual cleft tight enough to require manual distraction in order to visualize the base, and the wound or primary sinus tract opening was in this residual cleft. There were instances in which the residual cleft had existed since the cleft lift was performed but also instances where a new fold developed sometime after the procedure, as documented by the routine series of photos taken before and after all operations. The revision was offered at any time after the initial cleft lift if it was felt that it could speed up healing of a large dehiscence, if ultimate healing of a wound was doubtful, or if there was suspicion of a recurrent sinus.

Of the 76 revisions, 73 (96.1%) were successful. Three patients had a persistent tear of the anoderm, in a small fold, in the midline which required a second revision that rotated the area of the tear away from the midline and flattened the fold. This second revision was successful in all three of these patients. Therefore, all 76 patients ultimately went on to complete healing. The most common complication was a superficial, 1-2 mm, separation of the lower curve of the incision, for a maximum of 3 cm which healed by the eighth postoperative week with minimal wound care required by the patient.

Complications in the immediate post-op period included one patient with purulent drainage through the incision which required additional antibiotics and one patient with bleeding which was successfully evacuated by the previously placed drain. One patient developed an infected seroma four months postoperatively which was drained surgically. In no patients was there flap necrosis, nor inadvertent entry into the anal canal (Table 3).

**Table 3:** Complications of the cleft lift revision.

	Number	Percent
Ultimate successful healing	73	96.1
Required a second revision	3	3.9
Minor wound separation	18	23.7
Wound infection	1	1.3
Seroma	1	1.3
Hematoma	1	1.3

Because follow-up was so long in many of these patients, we had a chance to observe the long-term sequelae of the cleft lift revision, keeping in mind that the revision was often preceded by several other operations. Five patients indicated that they had some intermittent, mild, discomfort with sitting; these patients had all been subjected to wide excisions and/or failed cleft lifts before the revision (Table 4). There were no instances of fecal incontinence.

**Table 4:** Long-term follow-up issues in cleft lift revision patients.

	Number	Percent
Healed and asymptomatic	71	93.4
Mild intermittent discomfort	5	6.6

When surveyed six (8%) patients indicated that they have been diagnosed with hidradenitis suppurativa (HS) either before or after the cleft lift revision. None of these were the three patients requiring a second revision in this series, nor were they the patients with short-term complications.

## Discussion

The optimal treatment of pilonidal disease has been a surgical dilemma for many decades. The chapter titled Pilonidal Sinuses and Cysts in “Christopher's Textbook of Surgery” from 1942 states: “The constant flow of new procedures in the literature is the result of failures following the use of surgical procedures previously advocated.” The accepted etiology of pilonidal disease in 1942 was the existence of a congenital defect consisting of a “caudal appendage of the medullary canal” which therefore erroneously informed the type of procedures advocated [5]. The author described three surgical treatments: open excision, closed excision, and the use of sclerosants. He personally advocated closed excision but admitted that the reported incidence of failure for that procedure ranged from less than 1% to 50%. Currently, there is a dichotomy among the world’s surgeons regarding the treatment of this disease and many have not yet deviated from open or closed excision as advocated in 1942 nor have they improved on its success rate [4,6-8]. Fortunately, others are performing more contemporary procedures which provide greater success.

Now that there is very strong scientific evidence that pilonidal disease is acquired, rather than congenital, and caused by a deep gluteal cleft, the more recently developed operations address the underlying etiology more precisely [1]. In particular, the cleft lift operation as described by Dr. John Bascom addresses the depth of the gluteal cleft as the primary abnormality, and success rates with the cleft lift operation are high, and reproducible [9-12].

The cleft lift procedure has several technical nuances and there are some slight differences between the techniques described by various experienced cleft lift surgeons [3,9-12]. However, even with an experienced surgeon, failures, and recurrences occur. A 2018 meta-analysis by Stauffer et al. demonstrates that the Bascom Cleft Lift and Karydakis operations have the lowest failure rates, calculating a 2.7% failure rate at 120 months in 16,349 patients accrued from RCTs and non-RCTs [4]. Dr. John Bascom’s series of 69 cleft lift patients published in 2007 resulted in five patients who required one or two revisions, and a series of 700 patients published by the author in 2021 resulted in 24 patients requiring one or two revisions to gain complete healing [3,9]. This leaves a small percentage of patients who need a second operation in order to gain control of the pilonidal disease. As more surgeons around the world are performing cleft lifts or Karydakys Flaps, there will be more patients who require revisions.

As surgeons, it is our responsibility to have a treatment algorithm for patients with pilonidal disease that ultimately ends in complete healing, such that the patient can put this problem behind them and resume life without pain, drainage, dressings, and wound care. Whether a surgeon begins the treatment of the patient with excision, sinus tract ablation, or a complex flap, it is imperative that there be a series of logical steps ending with a durable solution. Unfortunately, we see many patients in our pilonidal clinic who have been abandoned by their surgeon and relegated to an open-ended regime of wound care after the failure of one or more operations.

The cleft lift has been shown to be an excellent solution to failed operations for pilonidal disease [1-3]. However, if patients have a failure of a cleft lift there should be another step in the treatment algorithm that includes the technique described in this article. In this cohort, we have seen two types of failures. The first is the initial failure of the incision to heal, and the second is initial primary healing followed by recurrent pilonidal disease. In both cases, this was caused by a deep gluteal fold that remained or developed after the cleft lift procedure was performed. We found that the recurrent wounds or primary sinus tract openings were always positioned in a deep skin fold, in the midline, and in proximity to the anus. The goal of revision in these patients was to flatten the deep fold and bring the entire incision off-midline.

In some situations, it is possible to look back at photos of the cleft lift and suspect future failure, and other times these failures are not predictable. The factors that predispose to failure are incisions that are not adequately lateralized and configurations that result in a persistent midline fold. With both types of failure, the resulting wounds or primary sinus tract openings are always in the perianal region, and in our series, 34% were actually on the edge of the anus (i.e., touching or overlapping the edge of the anoderm), a significant factor described in a previous publication by the author [3].

The technique described in this report is reproducible and successful. It can be intimidating for a surgeon because it requires the removal of a significant amount of perianal skin, and this should not be attempted without mentoring. However, it is an important adjunct to the performance of the initial cleft lift procedure. Additionally, with case-specific modifications, this technique can be used to repair failures of other types of pilonidal operations. There are several unintuitive aspects to this procedure, one of which is that redundancy is more of an issue than tension; wound separation from tension has not been an issue with the judicious use of the technique described, but redundant skin can cause recurrence.

There were no instances of flap necrosis. The thickness of the flap is about 1 cm, or slightly less, and is not developed any farther from the midline than necessary; both factors contribute to flap viability. Overall, this is a very well-vascularized and robust flap. Infection rates are acceptable with the prophylactic antibiotic regimen used in this series.

When performing a revision after a previous cleft lift, the key area requiring adjustment is invariably in the lower third of the gluteal crease. Although this brings the excision close to the anus, we have found that with careful dissection entry into the anal canal is avoidable, and never occurred in this series. Although it would seem that keeping the skin incision as far away from the anus as possible would be a valuable goal, this series demonstrates that an incision in proximity to the anus heals remarkably well as long as it is off-midline, and the author found it to be a dramatic improvement compared to techniques which curve the incision away from the anus. This technique takes advantage of the unique properties of the redundant perianal skin which allows a significant degree of rotation without tension as demonstrated in Figure 13. This particular anatomic characteristic can only be utilized if one curves the incision toward the anus and ends the incision on the edge of the anoderm. Encountering the external anal sphincter during mobilization of the anal flap did not cause fecal incontinence in these patients as either an early or late complication. Sitting immediately postoperatively does not cause wound separation, and is actually encouraged to allow air circulation to the perianal portion of the wound. Hair removal regimens were not recommended in any patients in this series.

An unexpected finding which emerged as follow-up was collected on this cohort is the high incidence of HS. The prevalence of HS in the United States has been described as low as 0.00033%, and as high as 4.1%, yet 8% of this group had this diagnosis either before or after their failed surgery; at least double, if not much higher, than the general population [13]. It is unclear whether this is because these patients were initially misdiagnosed with the pilonidal disease when in fact, they had HS; whether HS predisposes to pilonidal disease; or that HS causes failure of pilonidal surgery. This is an area for future study.

## Conclusions

The cleft lift procedure for pilonidal disease has one of the highest success rates of any of the operations for pilonidal disease, however, there are occasional patients who develop failure or recurrence. This article describes a revisional technique that repairs a failed cleft lift and was 96.1% successful in our series. Complications of infection, seroma, or hematoma occurred rarely. In the unusual situation in which the revision fails, it can be repeated with subsequent excellent results, and all patients in this series went on to complete healing. How to perform these revisions is an essential part of treating pilonidal disease, and this procedure should be part of the armamentarium of the experienced pilonidal surgeon in order to deal with the occasional failures that do occur with the cleft lift procedure.
